# Non-producer multiple myeloma presenting with acute hyperammonemic encephalopathy: case report

**DOI:** 10.1186/s13000-022-01285-6

**Published:** 2023-01-04

**Authors:** Karina Verma, Tina Zhang, David Mueller, Julie Li, Vaishali Sanchorawala, Andrew Staron

**Affiliations:** 1 Department of Medicine, Boston University Chobanian & Avedisian School of Medicine and Boston Medical Center, Boston, MA USA; 2 Boston University Chobanian & Avedisian School of Medicine, Boston, MA USA; 3 Department of Pathology and Laboratory Medicine, Boston University Chobanian & Avedisian School of Medicine and Boston Medical Center, Boston, MA USA; 4 Section of Hematology and Medical Oncology, Boston University Chobanian & Avedisian School of Medicine and Boston Medical Center, Boston, MA USA

**Keywords:** Hyperammonemia, Multiple myeloma, Non-producer, MASS-FIX, Daratumumab, Venetoclax

## Abstract

**Background:**

Hyperammonemic encephalopathy (HE) is a rare and life-threatening complication of multiple myeloma, with underlying mechanisms that are not fully understood. In contrast to previously reported cases, most of which have been associated with IgG or IgA isotypes, we describe a patient with HE as the presenting symptom of non-producer multiple myeloma (NPMM).

**Case presentation:**

A 60-year-old man developed lethargy that progressed into coma. He was found to have an elevated ammonia level, despite normal hepatic function. He was diagnosed with HE secondary to NPMM, demonstrating 80% plasma cells without light chain expression in the bone marrow and absence of a monoclonal protein in the serum or urine, including by matrix-assisted laser desorption ionization time-of-flight mass-spectrometry (MASS-FIX). Myeloma-directed therapy with daratumumab, bortezomib, cyclophosphamide and dexamethasone successfully reversed his HE. At clinical relapse, he received salvage chemotherapy followed by venetoclax therapy, leading to a short period of neurological recovery.

**Conclusions:**

This case demonstrates that HE can occur in a patient with NPMM and challenges the mechanism suggested by limited prior studies; i.e., that excess ammonia in multiple myeloma arises from degradation of M-proteins. We postulate that the neoplastic plasma cells in NPMM have amplified amino acid metabolism, despite lacking detectable intracellular or secreted immunoglobulins.

## Introduction

Secreted monoclonal (M) immunoglobulins, or their constituent chains, are a hallmark of multiple myeloma (MM). In less than 3% of MM cases, there is no measurable M-protein by serum and urine protein electrophoresis or immunofixation. This entity is termed non-secretory multiple myeloma (NSMM) [1, 2]. The diagnosis of NSMM is predicated upon evidence of ≥10% plasma cell infiltration in the bone marrow (BM) or biopsy-proven plasmacytoma; MM-related organ damage (CRAB features; i.e., calcium elevation, renal insufficiency, anemia and bone lesions); and lack of M-protein in serum and urine [3]. Among cases of NSMM, few have no cytoplasmic immunoglobulin synthesis detected by immunohistochemistry (IHC) or in situ hybridization (ISH) of plasma cells. This subgroup is called non-producer multiple myeloma (NPMM). Since treatment response criteria for MM rely on surveillance of M-protein, determining the success of therapy can be challenging in NSMM [4].

Symptomatic hyperammonemia is a rare and poorly understood manifestation of secretory MM, with an in-hospital mortality rate of approximately 44–48% [5, 6]. Excess ammonia production is thought to be the byproduct of M-protein biosynthesis and metabolism. Herein, we illustrate an unusual case of hyperammonemic encephalopathy (HE) resulting from NPMM. The co-occurrence of these two uncommon entities impels us to re-examine the biology of NPMM. This case further highlights how prompt treatment can curtail the severe neurologic deficits associated with MM-related hyperammonemia, although clinical responses may be short-lived even with modern treatment approaches.

### Case presentation

**Fig. 1:**
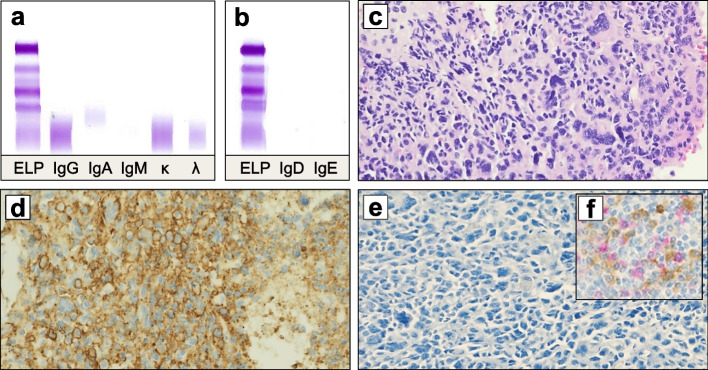
Absence of monoclonal band by **(a)** serum immunofixation electrophoresis, including antisera to **(b)** IgD and IgE. Bone marrow biopsy at the time of disease progression showing sheets of pleomorphic plasma cells by **(c)** hematoxylin and eosin stain at 20x; **(d)** highlighted by CD138 and **(e)** lacking cytoplasmic immunoglobulin expression by dual immunohistochemical staining of kappa and lambda with **(f)** control for comparison. *Abbreviation*: ELP, protein electrophoresis

A 60-year-old man with past medical history of coronary artery disease presented with back pain and intermittent confusion. His initial workup revealed hypercalcemia (serum calcium 14.8 mg/dL, normal range [NR] 8.6–10.3 mg/dL), acute renal failure (serum creatinine 5.0 mg/dL, NR 0.6–1.4 mg/dL) and hyperammonemia (serum ammonia 96 μmol/L, NR 18–72 μmol/L). Computed tomography (CT) imaging showed diffuse osteolytic lesions involving the calvarium. Serum immunoglobulin levels were low with IgG, IgA and IgM values of 473 mg/dL (NR 700–1600 mg/dL), 54 mg/dL (NR 70–700 mg/dL) and 19 mg/dL (NR 46–304 mg/dL), respectively. Measurement of serum free light chains showed a kappa level of 5.9 mg/L (NR 3.3–19.4 mg/L) and lambda level of 10.0 mg/L (NR 5.7–26.3 mg/L), with a kappa-lambda ratio of 0.59 (NR 0.26–1.65). Protein electrophoresis and immunofixation of the serum and urine, including antisera to IgD and IgE, detected no M-protein (Fig. 1 a, b). Matrix-assisted laser desorption ionization time-of-flight mass-spectrometry (MASS-FIX) was performed and found no M-protein in the serum. Note that serum MASS-FIX is a mass spectrometry-based method for detecting and isotyping serum M-proteins with higher sensitivity and specificity than immunofixation [7, 8]. BM biopsy showed 90% overall cellularity comprised of 80% CD138+ plasma cells with typical morphology, which were positive for cyclin D1 but lacked cytoplasmic expression of immunoglobulin kappa and lambda light chains by IHC and ISH. Cytogenetic analysis by fluorescence in situ hybridization (FISH) revealed the presence of translocation t(11;14), deletion of chromosome 13q, gain in chromosome 1q and trisomies of chromosomes 5, 9 and 15. All of these cytogenetic abnormalities are associated with MM. With a low serum albumin of 2.7 g/dL (NR 3.5–5.0 g/dL), high lactate dehydrogenase of 604 U/L (NR 171–308 U/L) and normal beta-2-microglobulin of 3.21 mg/L (NR 1.3–2.4 mg/L), he was diagnosed with Revised International Staging System (R-ISS) stage II NPMM.

**Fig. 2:**
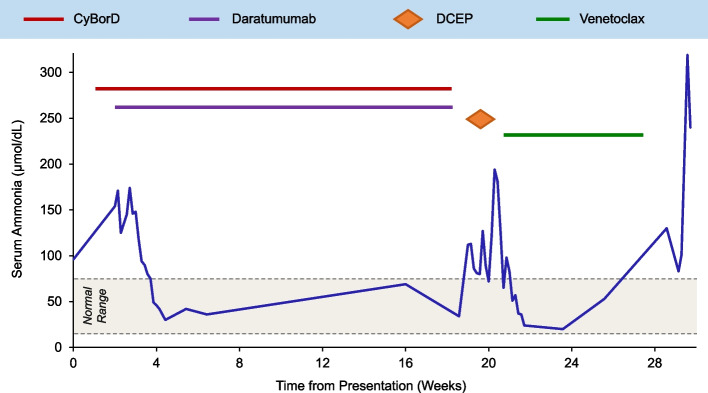
Serum ammonia concentration over the course of disease in relation to therapeutic interventions. Time zero is date of initial presentation. Normal range for ammonia: 8–72 μmol/L. *Abbreviations:* CyBorD = cyclophosphamide, bortezomib and dexamethasone; DCEP = dexamethasone, cyclophosphamide, etoposide and cisplatin

Despite normalization of serum calcium and renal function after isotonic fluids, the patient developed lethargy that progressed into coma over one week. Serum viscosity level was normal. Cerebrospinal fluid analysis, including a meningitis/encephalitis panel, was negative for infection or presence of myeloma cells. CT imaging showed cerebral edema, a potential effect of hyperammonemia, without other acute intracranial abnormalities or leptomeningeal disease. Serum ammonia concentration was now 199 μmol/L (Fig. 2). Having excluded other possible etiologies, his neurological condition was attributed to HE. Hepatic causes of hyperammonemia were ruled out with laboratory confirmation of preserved hepatic function, negative viral hepatitis serologies and normal liver structure on CT. Urinary orotic acid level was normal, thereby excluding an underlying ornithine transcarbamylase (OTC) deficiency. Hyperammonemia was concluded to be a direct consequence of NPMM. The patient was treated with rifaximin and lactulose, as well as extracorporeal dialysis for ammonia clearance, with minimal response. MM-directed therapy was initiated with once weekly cyclophosphamide IV at 225 mg/m^2^, bortezomib IV at 1.5 mg/m^2^ and dexamethasone IV at 40 mg (CyBorD). In order to more rapidly control hyperammonemia, the anti-CD38 monoclonal antibody daratumumab at 1800 mg SC was added on cycle 1 day 8. His serum ammonia concentration subsequently decreased to 42 μmol/L and encephalopathy improved by cycle 1 day 18.

Daratumumab plus CyBorD were continued post-hospitalization. Due to the lack of parameters for response assessment, BM biopsy was repeated after cycle 2 and demonstrated a partial response with 15% plasma cells remaining. During cycle 5, the patient re-presented with neurologic dysfunction from HE (serum ammonia 112 μmol/L). BM evaluation now revealed sheets of pleomorphic plasma cells lacking intracellular light chain expression (Fig. 1 c–e), indicating aggressive disease progression. Salvage therapy with dexamethasone IV bolus at 40 mg daily, plus cyclophosphamide at 400 mg/m^2^/day, etoposide at 40 mg/m^2^/day and cisplatin at 10 mg/m^2^/day as continuous IV infusion on days 1–4 (DCEP) was administered for one cycle. Considering the multi-refractory nature of his disease, rising ammonia level and positivity for translocation t(11;14), the B-cell lymphoma-2 (BCL-2) inhibitor venetoclax was started after DCEP, initially at a dose of 200 mg daily and later increased to 400 mg daily. While on single-agent venetoclax, he had a 5-week period of clinical response with reversal of encephalopathic state. He then again experienced neurologic deterioration as serum ammonia rose to 130 μmol/L, suggesting therapy resistance. The patient died of HE-related complications approximately 7 months after initial presentation.

## Discussion

This unusual case provides an opportunity to review hypotheses for the mechanisms of MM-related hyperammonemia. To our knowledge, the occurrence of hyperammonemia in NSMM has been reported only once before [9]. Upon closer scrutiny of that case, oligosecretory disease could not be ruled out, given the absence of comprehensive testing for trace levels of M-proteins undetectable by conventional methods. In our case, rare immunoglobulin isotypes of MM (i.e., IgD and IgE) were excluded and an advanced technique to evaluate for M-proteins below conventional detection thresholds was utilized. Lack of M-protein by serum MASS-FIX supported our diagnosis of NSMM. Absence of intracellular immunoglobulin kappa or lambda staining by IHC and ISH of BM plasma cells indicated non-producer subtype.

One proposed mechanism for hyperammonemia in MM is hepatic infiltration by malignant plasma cells, leading to liver failure or porto-systemic shunting [5, 9, 10]. Ammonia is largely produced within the gastrointestinal tract through the catabolism of nitrogen-containing compounds by bacteria, and subsequently processed by the liver [11, 12]. Endogenously generated ammonia from amino acid metabolism is also converted by the liver to urea, which is then excreted in urine by the kidneys. A hepatic etiology was unlikely in our patient, given the lack of liver pathology on imaging and laboratory studies. Furthermore, our patient did not respond to rifaximin and lactulose, both of which reduce production and absorption of ammonia in the intestine that is then processed by the liver. We inferred that hyperammonemia arose from direct overproduction by MM cells in the marrow, rather than a gastrointestinal or hepatic source.

An in vitro study previously demonstrated innate generation of ammonia by immunoglobulin-producing plasma cells [13] and provided an additional hypothesis for the mechanism of hyperammonemia in MM. Our patient’s NPMM may challenge this theory that excess ammonia arises from the degradation of M-proteins. Perhaps in our case, ammonia was derived from the catabolism of non-immunoglobulin proteins (e.g., cyclin D1 owing to t(11;14) translocation) or highly unstable immunoglobulin intermediates. Underlying urea cycle disorders can also be unmasked and lead to hyperammonemic crisis in conditions like MM with increased protein catabolism or periods of physiologic stress [14]. OTC deficiency is the most common inborn error of metabolism uncovered in adulthood. A normal urinary orotic acid level in our patient made this an improbable explanation for his HE.

For patients with MM-associated HE who survive the immediate risk period, little is known about their subsequent disease course. Clinical responses beyond 6 months from the start of treatment were observed only in few prior reports [15–17]. The longest documented response was a patient who remained neurologically intact after 18 months, despite experiencing moderate MM progression [18]. Most cases are associated with adverse prognostic risk (i.e., R-ISS stage III MM) and have an aggressive early disease course [5, 6, 19–22]. Our case had several high-risk features, including gain of chromosome 1q by FISH cytogenetic analysis and pleomorphic morphology. While HE can be a late manifestation of MM, in some cases like ours it is the initial presenting symptom, requiring early recognition and timely management [5, 9].

Prompt initiation of MM-directed therapy can effectively reduce serum ammonia levels [19], although the optimal approach and role of modern agents in this context remain unknown. Daratumumab-containing regimens are increasingly used in the frontline setting for MM [23, 24]. Interestingly, there has been at least one reported case of HE after the administration of daratumumab in a patient with relapsing kappa light chain MM [25]. Our patient was treated upfront with CyBorD in combination with daratumumab, providing a short-lived clinical response lasting approximately 4 months. For patients harboring chromosomal translocation t(11;14), venetoclax demonstrates promising activity in terms of overall response rate and progression-free survival, both alone or in a combined regimen [26–28]. In our case, venetoclax was used in the relapsed/refractory setting; however, this agent did not overcome the high-risk features of his disease.

## Conclusions

We report a case of neurological deterioration secondary to HE in NPMM. Innate production by myelomatous plasma cells was the most likely source of ammonia in this case. This implies that NPMM is metabolically active with amplified protein catabolism, despite lacking a detectable M-protein. Patients with MM-associated HE should be treated as soon as possible after diagnosis, although this phenomenon is often associated with advanced stage and high-risk disease that is treatment-resistant. Long-term prognosis remains poor in spite of modern therapeutic agents.

## Data Availability

Data sharing is not applicable to this article as no datasets were generated or analyzed during the current study.

## Abbreviations

BCL-2: B-cell lymphoma-2
BM: Bone marrow
CRAB: Calcium elevation, renal insufficiency, anemia, bone lesions
CT: Computed tomography
CyBorD: Cyclophosphamide, bortezomib and dexamethasone
DCEP: Dexamethasone, cyclophosphamide, etoposide and cisplatin
FISH: Fluorescence in situ hybridization
HE: Hepatic encephalopathy
IHC: Immunohistochemistry
ISH: In situ hybridization
M: Monoclonal
MASS-FIX: Matrix-assisted laser desorption ionization time-of-flight mass-spectrometry
MM: Multiple myeloma
NPMM: Non-producer multiple myeloma
NR: Normal range
NSMM: Non-secretory multiple myeloma
OTC: Ornithine transcarbamylase.
